# SYNTAX score effect on electroencephalography power dynamics in patients undergoing on-pump coronary artery bypass grafting

**DOI:** 10.1186/1471-2202-14-95

**Published:** 2013-09-06

**Authors:** Irina V Tarasova, Roman S Tarasov, Olga A Trubnikova, Olga L Barbarash, Leonid S Barbarash

**Affiliations:** 1 Department of Cardiovascular Diagnostics, Research Institute for Complex Issues of Cardiovascular Diseases, Russian Academy of Medical Sciences, Siberian Branch, Kemerovo, Russian Federation; 2 Department of Multifocal Atherosclerosis, Research Institute for Complex Issues of Cardiovascular Diseases, Russian Academy of Medical Sciences, Siberian Branch, Kemerovo, Russian Federation; 3 Director of Research Institute for Complex Issues of Cardiovascular Diseases, Russian Academy of Medical Sciences, Siberian Branch, Kemerovo, Russian Federation; 4 Chief Scientist of Research Institute for Complex Issues of Cardiovascular Diseases, Russian Academy of Medical Sciences, Siberian Branch, Kemerovo, Russian Federation

**Keywords:** Electroencephalography, Brain damage, Theta rhythm power, SYNTAX score, On-pump coronary artery bypass grafting

## Abstract

**Background:**

The severity of angiographically assessed coronary artery disease may be the factor that influences the degree of brain damage during on-pump surgery. Modern technology such as computed electroencephalography (EEG) that is used to detect signs of brain damage could also be used to determine the advantages and disadvantages of various surgical myocardial revascularization methods in certain categories of patients. The present study investigated EEG power dynamics for 1 postoperative month in patients undergoing on-pump coronary artery bypass grafting (CABG) who were divided into two groups: those with moderate coronary lesions (SYNTAX score ≤ 22, *n* = 12) and those with severe coronary lesions (SYNTAX score ≥ 23, *n* = 18).

**Results:**

At 7–10 days after CABG, all patients showed theta type 1 rhythm power higher than that seen preoperatively, possibly indicating that brain damage occurred during bypass. At 1 month after CABG, the theta type 1 rhythm power had decreased to the baseline level in patients with SYNTAX scores of ≤22, whereas it had increased in patients with SYNTAX scores ≥23.

**Conclusions:**

SYNTAX scores ≥ 23 are associated with EEG markers of perioperative brain damage during CABG. Careful preoperative assessment, preparation, and more effective intraoperative brain protection are essential for this category of coronary heart disease (CHD) patients.

## Background

Ischemic brain damage, a frequent complication of cardiac on-pump surgery, has been associated with postoperative stroke, encephalopathy, and neurocognitive dysfunction. It is established that these neurological complications can minimize the operation’s success, leading to social exclusion, impaired quality of life, and death during the long-term period after CABG [1,2]. The duration of cardiopulmonary bypass (CPB), manipulation of the aorta, and the effects of anesthesia have been suggested as causative factors in the development of cerebral ischemia during on-pump surgery. Preoperative factors, such as a high functional class of heart failure and angina, can also be predictors of postoperative neurological complications [3,4]. The severity of coronary artery disease may be associated with the number of grafts, manipulation of the ascending aorta, CPB duration, and the intensity of the systemic inflammatory response during the postoperative period—each of which can influence the degree of ischemic brain damage during cardiac surgery.

Currently, there are a number of scales that can objectively evaluate the complexity of coronary artery disease on the basis of angiographic data (SYNTAX score) or combined clinical and angiographic data. The latter include the Functional SYNTAX score, the New Risk Classification (NERS), and the Clinical SYNTAX score (CSS) [5-7]. In the literature, however, there is no evidence that these scales are of any predictive value with respect to neurological complications in patients undergoing CABG.

A widely discussed issue is the monitoring of ischemic brain damage due to CPB. Identifying minimal or subclinical signs of brain dysfunction using modern advanced technology can help determine the advantages and disadvantages of the various surgical revascularization methods in specific categories of patients. Multichannel computed EEG can be used to monitor brain electrical activity and thus detect signs of ischemic brain damage that can result in postoperative neurological deficit [8]. The noninvasiveness and simplicity of this method makes it possible to observe the dynamics of brain function recovery during the postoperative period [8,9]. A statistically significant relation has been found between the characteristics of intraoperative EEG and cognitive deficits for 2–3 months after CPB surgery [10]. Most of the association between cerebral hypoperfusion and EEG changes was identified during carotid surgery [10,11].

The aim of this study was to evaluate EEG power dynamics for 1 postoperative month in patients undergoing on-pump CABG. We relied on the SYNTAX score to indicate the severity of angiographically assessed coronary artery disease.

## Methods

### Subjects

All patients gave informed consent to participate in a prospective study aimed at studying changes in cognitive function after CABG. The Ethics Committee of Research Institute for Complex Issues of Cardiovascular Diseases, Russian Academy of Medical Sciences, Siberian Branch approved the study design.

Exclusion criteria were age > 70 years, initial depressive symptoms identified by the Beck Depression Inventory, dementia [sum score of the Mini-Mental State Examination ≤ 24], and the Frontal Assessment Battery score ≤ 11. Also excluded were subjects with a known history of rhythm disturbances, heart failure functional class IV according to the New York Heart Association (FC NYHA IV), concomitant diseases (chronic obstructive pulmonary disease, malignant pathology), diseases of the central nervous system, any episodes of a cerebrovascular accident, and/or brain injury. All patients were examined by a neurologist and underwent multi-spiral computed tomography during the preoperative period to detect any nervous system abnormalities. Only right-handed patients were included in the study so as to take account functional brain asymmetry.

A total of 30 male patients were divided into two groups: those with a moderate coronary lesion (SYNTAX score ≤ 22, *n* = 12, mean age 57.9 ± 5.47 years) and those with a severe coronary lesion (SYNTAX score ≥ 23, *n* = 18, mean age 57.5 ± 5.60 years). The severity of the coronary lesions was assessed using the results of coronary angiography and a SYNTAX calculator (http://www.syntaxscore.com/calc/start.htm). Before surgery the two groups were comparable with regard to a history of CHD, severity of heart failure (FC NYHA), ejection fraction (EF) of the left ventricle, neurological status, depression and anxiety score (Table 1). The degree of internal carotid artery (ICA) stenosis did not exceed 50% in any of the patients. Atherosclerotic stenoses in most of the patients were located at the ICA ostium.

**Table 1:** Demographic and clinical characteristics of the groups with different SYNTAX scores

**Characteristic**	**SYNTAX** ≤ **22 (n = 12)**	**SYNTAX** ≥ **23 (n = 18)**	**p**
Age (years)	57,9 ± 5,47	57,5 ± 5,60	>0,05
EF, %	57,9 ± 8,45	54,9 ± 12,44	>0,05
History of CHD, years	3,0 ± 4,78	4,8 ± 4,51	>0,05
FC NYHA, (%)			
II	75%	83%	>0,05
III	25%	17%	
Stenoses ICA, (%)	25%	39%	>0,05
Diabetes mellitus, (%)	33%	28%	>0,05
MMSE, (scores)	27,9 ± 1,58	27,3 ± 1,36	>0,05
FAB, (scores)	16,4 ± 1,03	16,2 ± 0,97	>0,05
Beck, (scores)	3,0 ± 1,61	2,75 ± 1,81	>0,05
State anxiety, (scores)	38,7 ± 5,45	39,6 ± 5,58	>0,05
Trait anxiety, (scores)	24,9 ± 10,66	20,2 ± 5,71	>0,05
CPB, min	117,1 ± 32,54	103,7 ± 22,54	>0,05
Aortic cross-clamping time, min	74,2 ± 20,15	70,1 ± 20,55	>0,05
Grafts, (n)	2,5 ± 1, 17	2,8 ± 0,53	>0,05

*CHD* coronary heart disease, *FC NYHA* functional class of heart failure by New York Heart Association, *EF* ejection fraction, *ICA* internal carotid artery, *MMSE* Mini Mental State Examination, *FAB* Frontal Assessment Battery, *Beck* Beck Depression Inventory Scale, *CPB* cardiopulmonary bypass.

All patients were treated before and after surgery with basic, symptomatic therapy according to the general principles of treatment for patients with CHD, heart failure, and hypertension (National Guidelines, 2009, 2008): low-salt (<1 g/day) and low-cholesterol diet, β-blockers (bisoprolol fumarate), angiotensin-converting enzyme inhibitors (enalapril maleate), and statins (rosuvastatin). Standard anesthesia and perfusion were carried out: combined endotracheal anesthesia (diprivan, fentanyl, sevoflurane). For all patients, CABG surgery was performed with normothermia. The average number of grafts, mean bypass time, and aortic cross-clamping did not differ between the two groups (Table 1). Invasive hemodynamic monitoring, carried out during the operation, indicated that there were no episodes of hypotension. Also, oxygenation of the cerebral cortex (rSO_2_) in real time was monitored (INVOS 3100; Somanetics, Troy, MI, USA) at all stages of the surgery. Brain hypoxia was not observed.

### EEG recording and processing

All EEG studies were performed at three time points: 3–5 days before surgery and 7–10 days and 1 month after surgery. High-resolution monopolar EEG recordings (62 channels, bandwidth 0.1–50.0 Hz) with closed eyes (EC) and open eyes (EO) were made in patients with moderate and severe coronary lesions. The EEG was amplified by Neuvo electroencephalography (Compumedics, Charlotte, NC, USA) using a modified 64-channel cap with sintered Ag/AgCl electrodes (QuikCap; Neurosoft, El Paso, TX, USA) according to the international 10–20 system. A reference electrode was attached to the tip of the nose and a ground electrode to the center of the forehead. Electrode resistance was maintained at <20 kΩ. The EEG was sampled at a rate of 1000 Hz over approximately 10 minutes. The data were analyzed offline. Visual inspection for eye movements, electromyographic interference, and other artifacts was undertaken. Artifact-free EEG fragments were divided into 2-s epochs and underwent Fourier transformation. For each subject, EEG power values were averaged within the delta (0–4 Hz), theta type 1 (4–6 Hz), theta type 2 (6–8 Hz), alpha type 1 (8–10 Hz), alpha type 2 (10–13 Hz), beta type 1 (13–20 Hz), and beta type 2 (20–30 Hz) bands. The mean values of the total EEG power in each band in the two states, EC and EO, were obtained. The data were log-transformed to normalize the distribution.

### Statistical analysis

The software package STATISTICA 6.0 (StatSoft, Tulsa, OK, USA) was used for all analyses of the variables. Categorical clinical data were analyzed with the χ^2^ Pearson adjusted Yates test. Quantitative measurements were done with the Wilcoxon and Mann–Whitney tests. Repeated-measures analysis of variance (ANOVA) was used to determine the difference in EEG power with Greenhouse–Geisser as correction. In each frequency range, the mean values of the total EEG power were analyzed to extract factors: SYNTAX (patients with SYNTAX score ≤ 22 or SYNTAX score ≥ 23), TIME (before CABG and at 7–10 days and 1 month afterward) separately for the EC and EO states.

## Results

We did not observe any life-threatening cardiac arrhythmias, acute coronary insufficiency with progression of heart failure, cerebral accidents, or transient ischemic attacks in any of the patients during the early postoperative period after CABG. A statistically significant EF decrease was found 7–10 days after CABG compared with the patients’ preoperative data. In patients with SYNTAX scores ≤ 22, the EF decreased from 59.9 ± 5.11% to 52.6 ± 6.56% (*p* = 0.003), and in those with SYNTAX scores ≥23 it decreased from 56.1 ± 12.15% to 49.3 ± 7.82% (*p* = 0.008). At 1 month, the EF had increased in all of the patients and did not differ from their baseline EFs.

**Figure 1:**
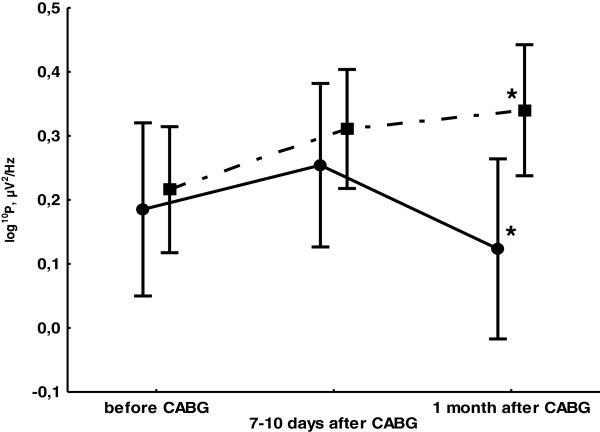
**Dynamics of theta type 1 rhythms mean power in the eyes-closed state separately for the SYNTAX scores** ≤**22 group (continuous line) and the SYNTAX scores** ≥**23 group (broken line).** Vertical bars denote 0.95 confidence intervals. Asterisks (*) indicate statistically significant differences (p < 0.05) between two groups.

ANOVA showed that theta type 1 (theta1) power is a significant main effect of TIME (F_2,56_ = 5.08, *p* = 0.015) and the interaction of SYNTAX–TIME (F_2,56_ = 6.15, *p* = 0.007). Patients with SYNTAX scores ≥23 had increased theta1 rhythms power with EC 7–10 days after CABG compared with baseline (*p* = 0.009). These changes remained at the 1-month follow-up (*p* = 0.00006). In patients with a SYNTAX scores ≤22, there was also a theta1 power increase 7–10 days after surgery (*p* = 0.035), but it had returned to baseline at 1 month (*p* = 0.03) (Figure 1). Patients with SYNTAX scores ≥23 had greater theta1 rhythm power than was seen in the group with SYNTAX scores ≤22 at 1 month after surgery (p = 0.04).

The analysis of theta1 rhythm power with EO revealed a significant effect of TIME (F_2,56_ = 10.4, *p* = 0.0004). The dynamics of theta power was similar in patients with moderate and severe SYNTAX scores, manifesting as a power increase 7–10 days after CABG (*p* = 0.0001) with a subsequent decrease to near baseline at the 1-month follow-up (*p* = 0.02).

A significant effect of TIME was found in theta type 2 (theta2) rhythm power with both EC and EO (F_2,56_ = 4.96, *p* = 0.01 and F_2,56_ = 10.2, *p* = 0.001, respectively). The rhythm power also increased 7–10 days after CABG (*p* = 0.008) and remained unchanged at 1 month.

Common effects (significant factor TIME, see Table 2) for all patient groups were found in alpha types 1 and 2 (alpha1,2) and beta types 1 and 2 (beta1,2) frequency ranges with both EC and EO 1 month after CABG. The alpha2 and beta1,2 rhythm powers with EC increased over baseline values 1 month after CABG (*p* = 0.0002, *p* = 0.0003, and *p* = 0.0007, respectively). With EO, the alpha1,2 and beta1 activity was increased 7–10 days after CABG with subsequent nonsignificant decreases to near baseline at the 1-month follow-up (*p* = 0.01, *p* = 0.0003, and *p* = 0.002, respectively).

**Table 2:** TIME effects on alpha types 1,2 and beta types 1,2 frequency ranges with EC and EO states

**EEG band/State**	**F**	**p**
Alpha types 1 /EO	9.52	0.0005
Alpha types 2 /EC	8.48	0.0007
Alpha types 2 /EO	10.64	0.0002
Beta types 1 /EC	7.39	0.0015
Beta types 1 /EO	15.01	0.00001
Beta types 2 /EC	4.4	0.02

**Figure 2:**
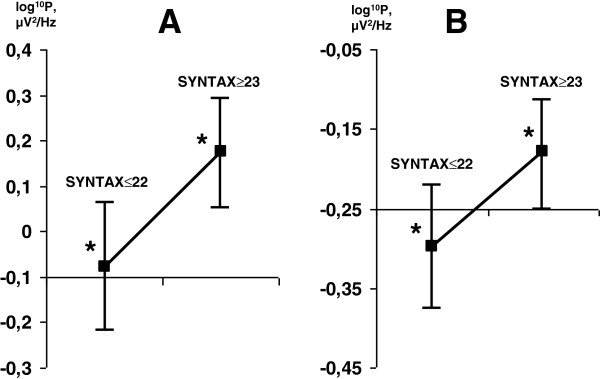
**Differences of alpha2 *(A) *and beta1 *(B) *rhythm mean power values in the eyes-open state between two groups with different SYNTAX scores.** Vertical bars denote 0.95 confidence intervals. Asterisks (*) indicate statistically significant differences (p < 0.05) between two groups.

The SYNTAX score was a significant factor (F_1,28_ = 7.04, *p* = 0.01) in alpha2 with EO and in beta1 (F_1,28_ = 9.08, *p* = 0.005) with both EC and EO (F_1,28_ = 6.18, *p* = 0.02). The alpha2 and beta1 rhythm powers were greater in the group with SYNTAX scores ≥23 (Figure 2).

## Discussion

We examined EEG power changes after CABG in patients with moderate and severe coronary lesions assessed by the SYNTAX score at 1 month of follow-up. We found that all patients demonstrated a higher power of theta1 and theta2 rhythms 7–10 days after CABG compared with preoperative data. In patients with SYNTAX scores ≥23, a significant low-frequency cortical activity increase continued even 1 month after surgery.

According to the literature, an increase of low-frequency activity in the resting state EEG, or “EEG slowing”, can be considered a marker of ischemic brain damage [8,10]. Brain damage may result from hypoperfusion and microembolisms during CPB [12], and this effect may continue after surgery. Extracranial vascular lesions may contribute to a chronic form of brain ischemia. In our previous study, we showed deteriorated neurological status of patients after CABG, especially in those with ICA stenoses [13]. In the present investigation, we found that a large number of patients with SYNTAX scores ≥23 had ICA stenoses (39% vs. 25%).

We assumed that the reason for prolonged EEG slowing after CABG is the increased expression of atherosclerotic changes in cerebral arteries of patients with SYNTAX scores ≥23. It has been shown that the severity of coronary atherosclerosis is directly correlated with extracranial and cerebral vascular lesions, in which case the risks of ischemic complications during CPB are dramatically increased [14,15]. Furthermore, severe coronary artery disease can be associated with longer, more traumatic effects on the ascending aorta during surgery [16]. We also found that patients with SYNTAX scores ≥23 had a larger number of grafts than did those with SYNTAX scores ≤22 (2.8 vs. 2.5).

The patients with SYNTAX scores ≥23 also had higher alpha2 and beta1 rhythm power values in the EO state before and after surgery. It is widely postulated that an increase in alpha power may reflect decreased cortical activation, whereas an increase in beta1 power is an unambiguous pathological sign of cortex dysfunction [8,17]. Thus, the group with SYNTAX scores ≥23 demonstrated signs of chronic brain ischemia preoperatively.

## Conclusions

A high SYNTAX score is associated with EEG markers of brain damage after on-pump CABG at the 1-month follow-up. The SYNTAX score may thus be an indirect indicator of involvement of other vascular zones, particularly cerebral arteries—a critical bit of information for the CPB surgeon. Careful preoperative assessment, preparation, and more effective intraoperative brain protection are essential for this CHD patient category. Also important is a consideration of safer surgery.

The SYNTAX score as an objective indicator of the severity of coronary artery disease can probably be integrated with other clinical factors: poor left ventricular contractility, diabetes mellitus, multi-vessel atherosclerotic lesions, and others that contribute to perioperative brain damage.

### Limitations of the study

It must be noted that this report is of a pilot study that was performed on a small set of patients (*n* = 30). Perhaps in this context, we have not demonstrated the complex relations of other clinical factors and their combined effect on the EEG markers of brain damage. These issues require further study.

Overall, our research has touched on neuropsychological, neurological, cardiac, and pathophysiological aspects of cardiac surgery, allowing a clear understanding of the mechanisms of neurological complications after CABG. Our findings can contribute to the search for optimal algorithms for preoperative evaluation of patients as well as the development of adequate revascularization and neuroprotection techniques.

## Abbreviations

EEG: Electroencephalography; CABG: Coronary artery bypass grafting; CHD: Coronary heart disease; CPB: Cardiopulmonary bypass; FC NYHA: Functional class of heart failure by New York Heart Association; EF: Ejection fraction; ICA: Internal carotid artery; EC: Closed eyes; EO: Open eyes.

## Competing interests

In the present study, there are no any actual or potential conflicts including any financial, personal, or other relationships with other people or organizations. The authors’ institution has no contracts relating to this research through which it or any other organization may stand to gain financially now or in the future. Authors or their institutions have no financial interest in this work. This manuscript has not been published elsewhere and is not under review with another journal. The present investigation adhered to the tenets of the Declaration of Helsinki and was approved by the institutional human experimentation committee. Informed consent was obtained from all subjects after the nature of the procedure had been explained. All authors have reviewed the contents of the manuscript being submitted, approve of its contents, and validate the accuracy of the data.

## Authors’ contributions

IVT and OAT designed the experiments. IVT performed the acquisition of the EEG data, statistical analysis and wrote the manuscript. RST and OAT performed the acquisition of the clinical data. OLB and LSB conceived the idea and edited the manuscript. All authors read and approved the final manuscript.
